# COVID-19 in the Americas and the erosion of human rights for the poor

**DOI:** 10.1371/journal.pntd.0008954

**Published:** 2020-12-18

**Authors:** Peter J. Hotez, Jorge A. Huete-Perez, Maria Elena Bottazzi

**Affiliations:** 1 Texas Children’s Center for Vaccine Development, Departments of Pediatrics and Molecular Virology & Microbiology, National School of Tropical Medicine, Baylor College of Medicine, Houston, Texas, United States of America; 2 Department of Biology, Baylor University, Waco, Texas, United States of America; 3 Hagler Institute for Advanced Study at Texas A&M University, College Station, Texas, United States of America; 4 James A Baker III Institute for Public Policy, Rice University, Houston, Texas, United States of America; 5 Scowcroft Institute of International Affairs, Bush School of Government and Public Service, College Station, Texas, United States of America; 6 Molecular Biology Center, University of Central America, Managua, Nicaragua; University at Buffalo - The State University of New York, UNITED STATES

*COVID-19 has emerged as a major neglected tropical disease in the Americas, so far responsible for 625,000 deaths. The illness has acquired a troubling and ominous human rights dimension*.

## Introduction

**Fig 1:**
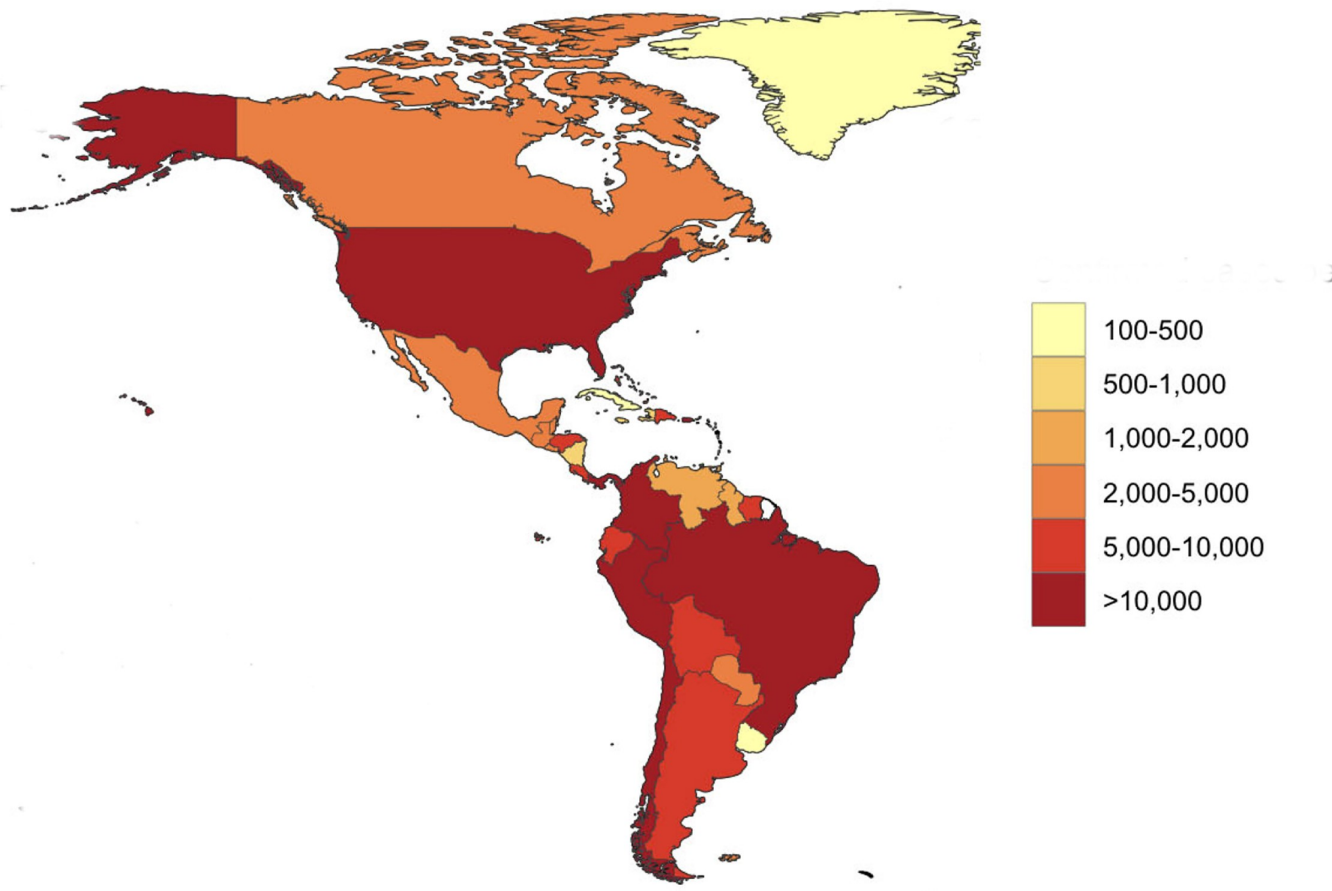
Heat map of coronavirus cases in the Americas. Cases per million people by country. Data source: https://github.com/owid/covid-19-data/tree/master/public/data, accessed August 31, 2020. Map template: https://www.naturalearthdata.com/downloads/50m-cultural-vectors/50m-admin-0-countries-2/.

Throughout the summer of 2020, the Coronavirus Disease 2019 (COVID-19) accelerated across Central and South America and the Caribbean. According to the World Health Organization (WHO) and its regional office in the Americas, the Pan American Health Organization (PAHO), as of August 2020, more than one-half (54%) of the world’s COVID-19 cases and most of the deaths (75%) have occurred in the Americas, led by the United States and Brazil as the 2 most affected countries, followed by Colombia, Peru, Argentina, and Mexico (Fig 1) [1].

Increasing evidence indicates that those living in low-income neighborhoods in the Americas are disproportionately affected by COVID-19, although the basis of linking poverty to illness requires further elucidation. Among the reasons commonly cited are the difficulties of social distancing in crowded urban areas [2], lack of access to healthcare or public health messaging, requirements for essential workers to continue employment in high-risk settings, the particular susceptibilities of indigenous populations [3], and the high rates of underlying non-communicable diseases among the poor, including diabetes and hypertension [4]. Based on these observations and links to extreme poverty and related social determinants of health [5], we and others have noted how COVID-19 exhibits many of the features common to neglected tropical diseases (NTDs) [6] and the potential for COVID-19 to disrupt current NTD control and other disease intervention activities [7]. The NTD link for COVID-19 is especially relevant to the framework of “blue marble health” which finds that many if not most NTDs found in the Americas affect the poor living in the US, Brazil, Argentina, and Mexico [8].

### Neglected diseases, human rights, and populism

Is there a human rights dimension to COVID-19 in the Americas? From 2002 to 2008, Professor Paul Hunt was designated UN Special Rapporteur on the right to health. At that time, new international and regional treaties were established under a framework of the “right to the highest attainable standard of health” [9], which coincided with a new health framework and access to essential medicines for NTDs [10,11]. Following a visit to Uganda, Hunt identified some key elements related to human rights for NTDs, including the need for overall strengthening of health systems, local needs, and urgency to incentivize local healthcare workers, dispelling myths through public information campaigns, destigmatizing NTDs, access to essential medicines and research and development (R&D), and monitoring and accountability [9]. Through discussions with the Government of Peru, another dimension was to facilitate access to innovations by incorporating human rights into trade agreements [7]. Simultaneously, Chris Beyrer and his colleagues at Johns Hopkins University noted how NTDs such as Chagas disease, leishmaniasis, and yellow fever reemerged in Colombia in association with guerilla movements and the drug trade that resulted in the systematic violations of human rights [12]. Both initiatives build on historically important documents and frameworks including the Universal Declaration of Human Rights (1948) and the International Covenant on Social, Cultural and Economic Rights (1976). Article 12 of the Covenant specifies: “The prevention, treatment and control of epidemic, endemic, occupational and other diseases” (https://www.ohchr.org/en/professionalinterest/pages/cescr.aspx). Also relevant are the Siracusa Principles on the Limitation and Derogation Provisions in the International Covenant on Civil and Political Rights (1984) and their relevance to quarantine ethics [13].

While so far the human rights dimension to COVID-19 in the Americas has no obvious overriding or unifying theme, it has been noted how populism or populism linked to far-right nationalism might fuel NTDs and therefore COVID-19 [14]. There are multiple definitions of populism, but typically the term refers to a political system that advocates for ordinary individuals and pitches them against the wishes of the elites. It arose in the late 1800s in the American Midwest by the formation of a US Populist Party that included farmers who felt excluded from the economic gains resulting from business interests or government corruption [15]. However, in this 21st century, populism has paradoxically also become attached to far-right interests. Ariel Pablos-Mendez, formerly at the Rockefeller Foundation and the United States Agency for International Development, has noted how this new brand of populism has promoted the emergence of COVID-19 through a consistent pattern of denial and neglect at the level of federal governments, leaving individual states or local municipalities in nations such as Brazil or Mexico to combat the pandemic [16]. This has also been observed in the US, where in place of federal directives each state was left to making their own decisions [17]. In these instances, populist-led federal governments either deny the seriousness of the COVID-19 pandemic or implicate conspiracies as the basis for its emergence of COVID-19. They rely on this misinformation to justify inaction or intervention against the disease at the federal level.

### Regional differences in the Americas

#### United States and Canada

In the US, as stated above, the federal response, or the lack of a federal response, has similarly proceeded along the lines outlined by Pablos-Mendez for the Latin American region. Through the White House Coronavirus Task Force under the leadership of US Vice President Mike Pence, except for some backup emergency support, manufacturing of protective equipment and ventilators, and supply-chain management, the efforts to contain the epidemic in the US have been mostly left to states or local authorities. As a result, the US accounts for approximately one-quarter of the pandemic in terms of new cases and a significant percentage of deaths [1]. Less commonly appreciated is the fact that in the US, COVID-19 has emerged as a health disparity, resulting in a disproportionate amount of illness, hospitalizations, and deaths among Hispanic, African-American, and Native American populations [18,19]. Tragically, these populations also constitute a much higher percentage of the deaths among young people and those under the age of 65 [20]. As the epidemic swept across the Southern US in the summer of 2020, it was noted how COVID-19 particularly affected Hispanic neighborhoods leading one of us (PJH) to refer to this situation as “historic decimation” of Hispanic communities in the US, especially in Texas metropolitan areas and in South Texas [21]. The impact of COVID-19 in US jail and prison populations has also been noted, and the fact that some of the largest individual outbreaks now occur among incarcerated individuals [22]. In both instances, the human rights aspects of NTDs outlined by Hunt applies, including heightened disease exposure in low-income neighborhoods and lack of access to healthcare or public health information. In contrast to the US, the disease burden from COVID-19 in Canada has been comparatively modest, although it is expected to rise considerably this winter.

#### Mesoamerica: Mexico and Central America

Similarly in Mexico, the populist regime of Andres Manuel Lopez Obrador has mostly left virus containment to the individual states [23]. Those at the greatest risk of death from COVID-19 in Mexico include males over the age of 41 with underlying conditions, including diabetes, hypertension, and obesity [24], especially those living in poverty [16]. In Central America, populist governments on opposing points of the political spectrum, such as Nicaragua and El Salvador, have rejected the opinions of scientists and experts, have failed to assuage misinformation, and have been incapable of designing and implementing well-defined, solid, and effective strategies to confront the COVID-19 pandemic, including the strengthening of national health systems and increasing access to adequate care. They have also failed to mitigate the social and economic impact of the disease on their respective populations. Nicaragua's government, led by President Daniel Ortega, has been criticized for its nonexistent response to COVID-19, resulting in a significant and preventable upsurge of cases [25,26], although COVID-19 is also widespread among more stable democracies in the region, including Panama. Early in the pandemic, the government of El Salvador adopted stringent measures, such as the closing of borders and involving forced isolation [27]; in contrast, its Nicaraguan counterpart not only ignored WHO recommendation for physical distancing measures [28], but even encouraged instead mass gatherings and keeping schools and businesses open even during the most acute months of the pandemic in terms of cases and deaths [29]. By ignoring the advice of scientists and experts, and placing political interests above scientific criteria, the governments of Nicaragua and El Salvador have also undermined detrimentally the role of science and scientists in both countries.

#### South America

Brazil accounts for the largest numbers of cases and its deaths may actually exceed those in the US but they are often not counted in official statistics. Unions, social organizations, and health professionals recently filed a complaint against populist Brazilian President, Jair Bolsonaro, to the International Criminal Court in The Hague, the Netherlands, for his neglect and denial in combating COVID-19, while more than 150 Catholic bishops penned a letter openly criticizing the President [30]. Both US President Trump and Bolsanaro have aggressively promoted the use of hydroxychloroquine for the treatment and prevention of COVID-19 despite evidence showing that it does not work and can be dangerous in high doses [30]. Moreover, some of the worst allegations against the Bolsonaro regime are directed against their refusal to take action to prevent COVID-19 among Brazil’s indigenous populations now being decimated by the illness [31,32].

Overall, in the Americas, countries such as Costa Rica and Uruguay stand out as regional success stories for having implemented a swift and effective pandemic response, which included timely detection and isolation of cases, shuttering schools, closing borders, and forbidding mass events. Among the decisive factors that helped these countries to contain the virus are their universal health system, solid healthcare infrastructure, and utilizing their noteworthy technological infrastructure and scientific talent to tackle the outbreak [33]. Furthermore, compared to its Latin American peers, both countries present unique advantages such as their higher level of development, good governance, and well-established democratic institutions.

### COVID-19 vaccines and biotechnology

A further human rights dimension is now emerging from lack of international access to COVID-19 vaccines, therapeutics, and diagnostics. For instance, a global initiative is underway to accelerate access to vaccines, and even though it is not clear which vaccines will be deployed, these rely on multiple different vaccine technologies under evaluation. The US-led activity known as Operation Warp Speed is also underway, albeit is not participatory in the global strategy. In the absence of US leadership, an ominous trend sometimes referred to “vaccinationalism” has emerged, which refers to vaccines by their nations of origin. This concept is in stark contrast to the promise of “vaccine diplomacy” intended to promote the sharing of vaccine development and distribution between nations [34].

Central to current efforts toward global vaccine access, and one adapted from previous Advanced Market Commitment (AMC) strategies, is the COVID-19 Vaccine Global Access (COVAX) Facility, led by Gavi, the Vaccine Alliance, in collaboration with WHO and CEPI, the Coalition for Epidemic Preparedness Innovations [35]. However, concerns remain in terms of whether this initiative will support LMICs especially in Latin America [36]. COVAX so far does not guarantee access to more than 20 percent of the populations including in Latin America. Even though Gavi has previous experience with similar mechanisms, in view of the magnitude of COVID-19 vaccine doses needed and their current cost-structure, the organization and the global governance of vaccine development and distribution is faced with unprecedented challenges. Hence, there are heartfelt worries that populations living in extreme poverty in the region won’t have vaccine access.

Such potential shortfalls have left the region of the Americas searching for alternatives, including bilateral initiatives to access vaccines including agreements leading to various dimensions of technology transfer to produce or condition vaccines in the region. An example is the one led by AstraZeneca with Argentina and Mexico [37]. Another is being accelerated through low-cost vaccine platforms focused on adopting proven vaccine technologies used for other global vaccines. For example, Texas Children’s Center for Vaccine Development and Baylor College of Medicine have partnered with Biological E Ltd in India to produce and advance a low-cost recombinant protein COVID-19 vaccine for global health [38]. This vaccine employs technology similar to the recombinant hepatitis B vaccine produced through microbial fermentation in yeast, with plans to also accelerate the vaccine for the Latin American region.

These urgencies to acquire or develop COVID-19 vaccines exposed deep weaknesses in the pandemic preparedness infrastructures for the Latin American region. Beyond vaccines, while the most industrialized countries in the Americas can support a range of new lab-based and point-of-care testing facilities, in the less developed countries, the potential to combat emerging and neglected diseases will require support and funding from regional development banks. This is especially true for developing mission-critical biotechnology infrastructure and strengthening the region for home-grown local production of vaccines, drugs, and diagnostics.

## Conclusions

COVID-19 in the Western Hemisphere has acquired a huge and ominous human rights dimension, especially among the poor and underserved populations living in the US, Mexico, and Central and South America. Populations living in poverty, especially indigenous people, have been placed at increased risk to COVID-19 compared to others, and there are grave concerns that such groups will also be denied access to preventative vaccines and medicines. Furthermore, as COVID-19 emerges as the newest serious NTD in the region leading to long-term morbidities, there is now an urgent need to convene the leadership of the Latin American nations. Near-term and long-term strategies must be shaped, and these can only be achieved with the direct involvement and support from the Organization of American States and PAHO-WHO, together with the Interamerican Development Bank, other regional banks, and the private sector. We are now at 625,000 deaths and counting in the region, but so far, an international response remains missing in action.
